# The food additive EDTA aggravates colitis and colon carcinogenesis in mouse models

**DOI:** 10.1038/s41598-021-84571-5

**Published:** 2021-03-04

**Authors:** Rayko Evstatiev, Adam Cervenka, Tina Austerlitz, Gunther Deim, Maximilian Baumgartner, Andrea Beer, Anita Krnjic, Christina Gmainer, Michaela Lang, Adrian Frick, Helga Schachner, Vineeta Khare, Christoph Gasche

**Affiliations:** 1 Division of Gastroenterology and Hepatology, Department of Internal Medicine III, Medical University of Vienna, Waehringer Guertel 18-20, 1090 Vienna, Austria; 2 Department of Pathology, Medical University of Vienna, Waehringer Guertel 18-20, 1090 Vienna, Austria

**Keywords:** Experimental models of disease, Translational research, Cancer prevention, Gastrointestinal cancer, Gastrointestinal diseases

## Abstract

Inflammatory bowel disease is a group of conditions with rising incidence caused by genetic and environmental factors including diet. The chelator ethylenediaminetetraacetate (EDTA) is widely used by the food and pharmaceutical industry among numerous other applications, leading to a considerable environmental exposure. Numerous safety studies in healthy animals have revealed no relevant toxicity by EDTA. Here we show that, in the presence of intestinal inflammation, EDTA is surprisingly capable of massively exacerbating inflammation and even inducing colorectal carcinogenesis at doses that are presumed to be safe. This toxicity is evident in two biologically different mouse models of inflammatory bowel disease, the AOM/DSS and the IL10^−/−^ model. The mechanism of this effect may be attributed to disruption of intercellular contacts as demonstrated by in vivo confocal endomicroscopy, electron microscopy and cell culture studies. Our findings add EDTA to the list of food additives that might be detrimental in the presence of intestinal inflammation, but the toxicity of which may have been missed by regulatory safety testing procedures that utilize only healthy models. We conclude that the current use of EDTA especially in food and pharmaceuticals should be reconsidered. Moreover, we suggest that intestinal inflammatory models should be implemented in the testing of food additives to account for the exposure of this primary organ to environmental and dietary stress.

## Introduction

Western diet and modern highly processed foods are believed to play a key role for the growing incidence of IBD and especially Crohn’s disease^1–3^, although the identification of specific noxious agents remains a challenge.

Chelators are molecules that form stable complexes with a single central ion, thereby keeping the ion in solution but suppressing its chemical activity. EDTA is the most commonly used chelator worldwide, with annual production exceeding 50,000 tons just in the European Union^4^. It finds application in the agriculture, chemical, textile and paper industry, as well as in cosmetics, household chemicals and medications. EDTA compounds are used in the food industry as sequestrants and stabilizing agents such as Na_2_EDTA·2H_2_O (Na-EDTA for short, E386), or CaNa_2_EDTA·2H_2_O (Ca-EDTA, E385) improving colour and flavor stability, or as a vehicle for iron fortification (FeNaEDTA·3H_2_O or Fe-EDTA). Due to the widespread use of EDTA and its high stability, EDTA compounds accumulate in nature and are ubiquitously present including detectable amounts in the drinking water^4^. Numerous studies in healthy animals have shown no considerable acute or repeat dose toxicity of EDTA^5,6^. Based on a no observed adverse effects level (NOAEL) of 250 mg/kg body weight (bw) for Fe-EDTA, international authorities recommend an acceptable daily intake (ADI) of maximum 1.9 mg EDTA/kg bw for humans^5,6^.

Here we show that EDTA compounds aggravate intestinal inflammation and colitis-associated carcinogenesis in two mechanistically different mouse models, at doses that are expected to be non-toxic according to current regulations and recommendations. EDTA disrupts various components of the intestinal barrier, providing a potential mechanism for the observed effects. Our results add EDTA to the growing list of food additives that may constitute an environmental factor for IBD and colitis-associated carcinogenesis.

## Results

**Figure 1:**
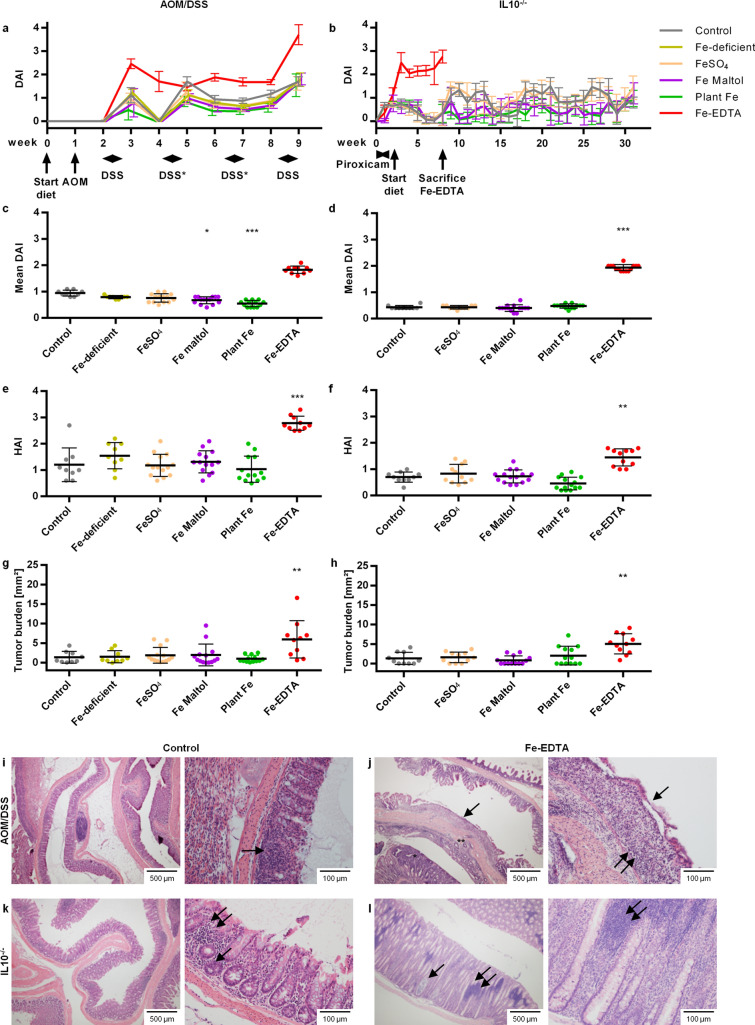
Fe-EDTA but not other iron compounds increase colitis activity and colorectal carcinogenesis in the AOM/DSS and IL10^−/−^ models of IBD. **(a**,**b)** Time course of the clinical disease activity index DAI in the AOM/DSS (**a**) and IL10^−/−^ (**b**) model. The timeline of experimental interventions is shown on the x axis. The omitted DSS cycles in the Fe-EDTA group due to high colitis activity are marked with an asterisk. (**c,d)** Mean DAI over the full time course (i.e. weeks 2–9 for AOM/DSS (**c**), weeks 1–8 for IL10^−/−^ (**d**)); (**e,f**) Histological activity index (HAI) for AOM/DSS (**e**) or IL10^−/−^ (**f**); (**g**,**h**) Tumour burden (i.e., total tumour area per mouse) for AOM/DSS (**g**) or IL10^−/−^ (**h**); exemplary image of hematoxylin–eosin-stained intestines of control (**i,k**) and Fe-EDTA-fed (**j,l**) animals. (**i**) DSS-induced increased inflammatory infiltrate (arrow) with partial loss of crypts in a control animal from the AOM/DSS model. (**j**) massive inflammation (double arrow) with complete crypt destruction and a single regeneratory layer of epithelial cells covering the lamina propria (single arrow) in an Fe-EDTA-treated animal from the AOM/DSS model. On the lower magnification image (left side), an invasive tumour (*) is seen; the point of invasion through the lamina mucularis mucosae is marked with **. (**k**) Inflammatory infiltrate (double arrow) and cryptitis through invading neutrophils (single arrow) in a control animal from the IL10^−/−^ model. (**l**) Marked hyperplasia, crypt abscess (single arrow) and massive inflammatory infiltrate (lymphocyte aggregates; double arrow) in an Fe-EDTA-treated animal from the IL10^−/−^ model. Error bars represent standard deviations. Asterisks (*: p < 0.05; **: p < 0.01; ***: p < 0.001) denote statistically significant results compared to the control group.

We were primarily interested in comparing iron compounds at a dose mimicking oral iron replacement therapy, for their potential to aggravate colitis and colitis-associated carcinogenesis. Several studies have implied a negative effect of oral iron replacement and associated high luminal iron concentrations in IBD^7–12^, although the effect in the human setting is controversial^13,14^. We examined the effect of oral iron compounds with different chemical properties on clinical and histological inflammation as well as on tumorigenesis in the azoxymethane—dextran sodium sulfate (AOM/DSS) mouse model^15,16^, as well as the interleukin 10-knockout (IL10^−/−^) mouse^17^. The selected compounds were: ferrous sulfate (the most commonly used ferrous salt for oral iron replacement), ferric maltol (a novel trivalent iron compound that has been licensed for iron replacement therapy in IBD^18^), plant iron (an extract from the curry leaf plant (Bergera koenigii)), and Fe-EDTA (a product with high bioavailability which is recommended by the World Health Organization as an iron fortificant^19^). Control animals received ferrous sulfate at 45 mg elemental Fe/kg chow, i.e. the iron content of the standardized rodent chow AIN76A. The test substances were mixed into an iron-depleted chow to achieve a tenfold iron content of AIN76A (Supplementary Table S1). We were surprised to find that only Fe-EDTA exacerbated colitis and massively increased tumour burden, while the other iron compounds did not differ from the control (Fig. 1, Supplementary Fig. S1). In the AOM/DSS-model, the mice fed Fe-EDTA were only able to tolerate the first and fourth DSS cycle due to massive weight loss and diarrhea. Similarly, in the IL10^−/−^ model, the Fe-EDTA group developed severe intestinal inflammation leading to premature sacrifice at week 8 compared to week 31 for the other groups.

**Figure 2:**
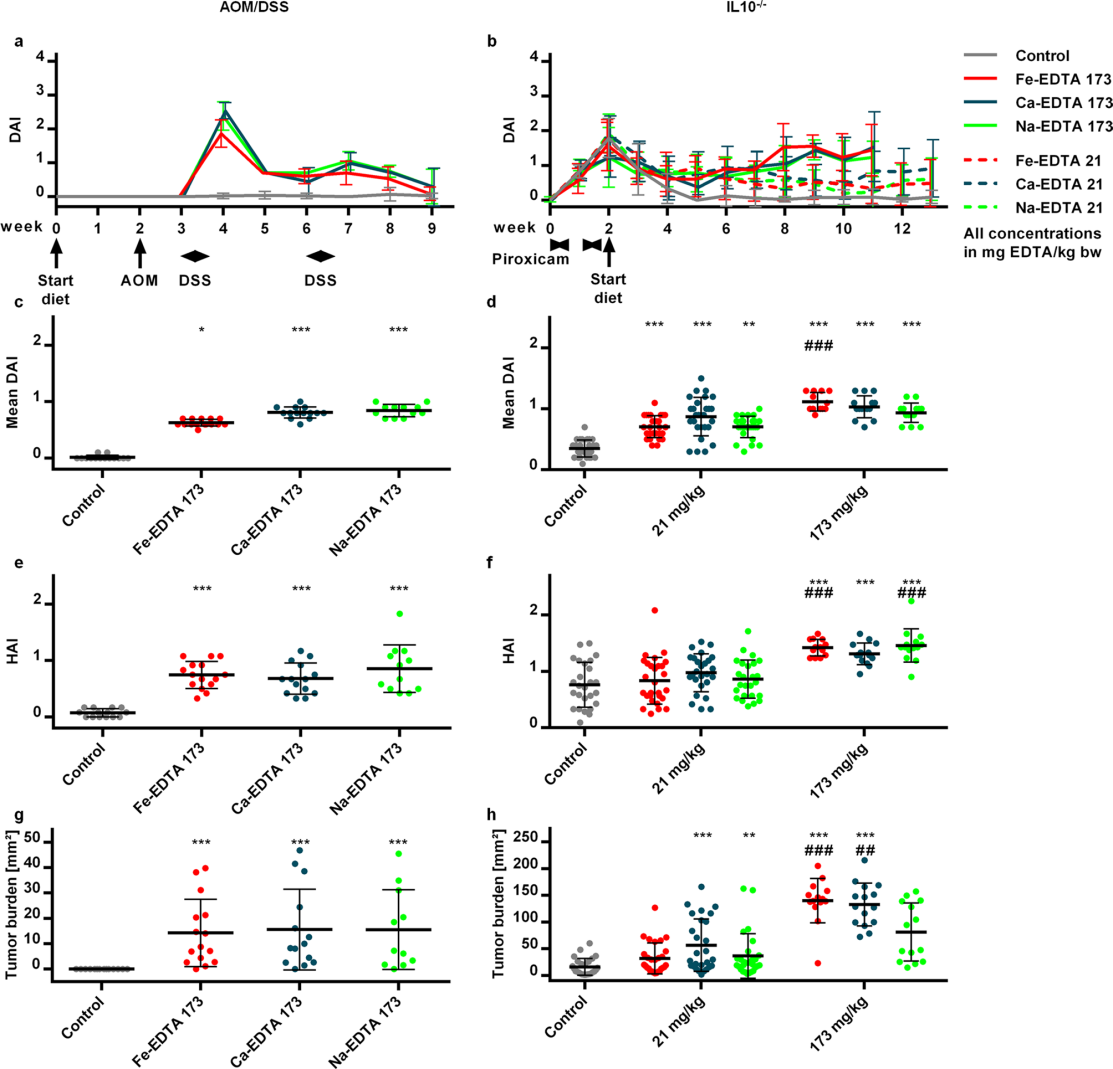
EDTA compounds enhance colitis activity and colorectal carcinogenesis in the AOM/DSS and IL10^−/−^ models.** (a**,**b) **Time course of the clinical disease activity index DAI in the AOM/DSS (**a**) and IL10^−/−^ (**b**) model. The timeline of experimental interventions is shown on the x axis. (**c,d**) Mean DAI over the full time course ((i.e. weeks 3–9 for AOM/DSS (**c**) and weeks 2–11 for IL10^−/−^ (**d**)); (**e**,**f**) Histological activity index (HAI) for AOM/DSS (**e**) or IL10^−/−^ (**f**); (**g,h**) Tumour burden (i.e., total tumour area per mouse) for AOM/DSS (**g**) or IL10^−/−^ (**h**). Error bars represent standard deviations. Asterisks (*: p < 0.05; **: p < 0.01; ***: p < 0.001) denote statistically significant results compared to the control group; hashtags (#: p < 0.05; ##: p < 0.01; ###: p < 0.001) mark significant differences between both EDTA compound doses.

Next we tested whether the intestinal toxicity is due to EDTA or is only specific to Fe-EDTA. We compared Fe-EDTA, Ca-EDTA and Na-EDTA added to a regular chow to a control group. Two EDTA doses, 173 mg EDTA/kg bw, corresponding to the NOAEL in rodents, and 21 mg EDTA/kg bw, mimicking the human ADI dose (Supplementary S1) were used. Also, a less aggressive treatment with longer recovery phases after DSS was applied. The results demonstrated that, in the AOM/DSS model, all EDTA compounds led to higher colitis activity compared to the control group (Fig. 2). No tumours were found in the control group compared to EDTA groups. Histological activity was low in all groups, partially because mice were sacrificed after 11 days of recovery from DSS. In the IL10^−/−^ model, the colitis activity was also higher in EDTA groups. A dose-related effect could be observed, with groups treated with 173 mg/kg having higher colitis activity than groups treated with 21 mg/kg. EDTA groups, especially those treated with 173 mg/kg also had a significantly higher tumour burden than the control group (Fig. 2, Supplementary Fig. S2). These results confirm that the aggravating effect on colitis and colitis-associated carcinogenesis was not specific to a certain EDTA compound but rather conferred by EDTA itself.

EDTA is a chelator with high affinity for di- and trivalent cations including Ca^2+^ and Mg^2+^. Many of the intracellular contacts are directly (cadherin-based contacts, i.e. adherens junctions (AJs) and desmosomes) or indirectly calcium-dependent (tight junctions (TJs) and integrin-based contacts, i.e. hemidesmosomes^20,21^). Of note, EDTA is widely used in biomedical research to detach adherent cultured cells or to isolate epithelia from organs^22,23^. Hence, we hypothesized that the toxic effect of EDTA may be conferred by disruption of the mucosal barrier and intercellular contacts. Therefore, we examined different components of the intestinal barrier (mucus layer, TJs, AJs and desmosomes) in the intestines from different groups of EDTA-treated animals. No changes were observed in expression of ZO-1 (marker of TJs) or desmoglein-2 (desmosomes); however, markers of AJs (β-catenin and E-cadherin) showed modestly altered expression. A loss of membranous β-catenin in distal colon (where disease activity was highest in AOM/DSS mice) was observed (Supplementary Fig. S3). E-cadherin also tended to reduced membranous expression with increased cytoplasmic localization. A similar trend was observed in IL10^−/−^ mice. These changes indicated weakening of AJs and cellular contacts. Overall, these observations were not conclusive about EDTA-specific effects in the setting of mild histological disease activity at the time of sacrifice as well as regenerative inflamed mucosa with hyperplastic and dysplastic tissue architecture.

**Figure 3:**
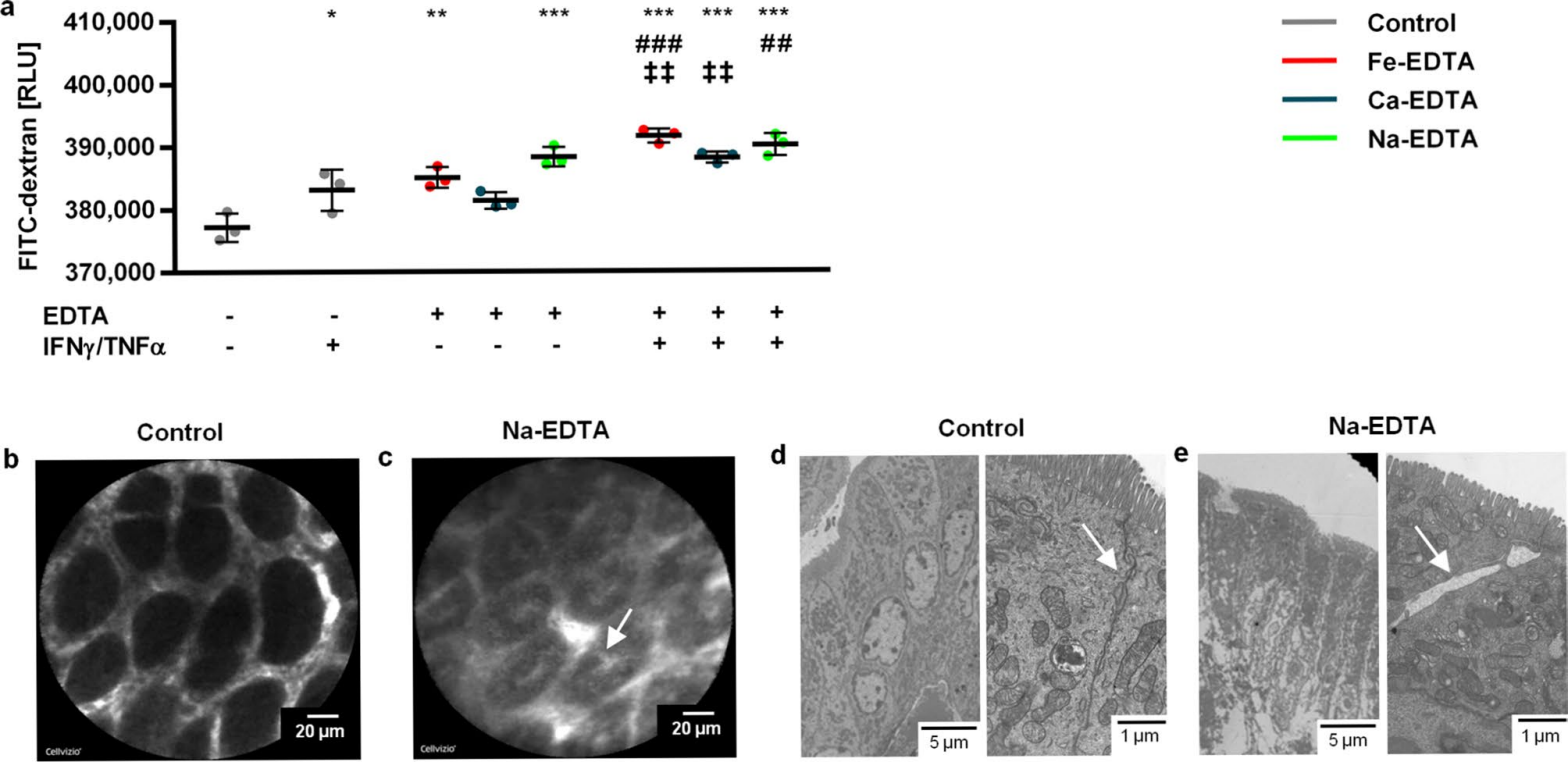
EDTA compounds increase paracellular permeability by damaging intercellular contacts. (**a)** Results of the in vitro FITC-dextran permeability assay on T84 cell monolayers exposed to EDTA compounds and/or IFNγ plus TNFα as noted. Error bars represent standard deviations. Asterisks (*: p < 0.05; **: p < 0.01; ***: p < 0.001) denote statistically significant results compared to the control group, hashtags (^#^: p < 0.05; ^##^: p < 0.01; ^###^: p < 0.001) mark significant differences compared to the IFNγ plus TNFα treated monolayers, and double daggers (^‡^: p < 0.05; ^‡‡^: p < 0.01; ^‡‡‡^: p < 0.001) indicate significant comparisons between EDTA compounds with or without IFNγ plus TNFα. (**b**,**c**) Confocal laser endomicroscopy of intestinal epithelium in healthy mice pretreated with Na-EDTA rectally (**c**) or sham treated (controls; **b**). The arrow shows accumulation of fluorescein in the crypt lumen and paracellular fluorescein plumes with Na-EDTA. (**d**,**e**) Transmission electron microscopy of intestinal tissues from healthy mice treated with Na-EDTA rectally (**e**) or controls (**d**). The arrow demonstrates gaps in the intercellular space indicative of breakage of intercellular contacts and specifically of AJs by Na-EDTA. The intercellular contacts in control animals are intact.

Intestinal inflammation itself is known to weaken the intestinal barrier^23,24^. In order to examine the EDTA effect independently of colitis, we exposed cultured monolayers of T84 cells to EDTA compounds. Also, to mimic inflammation, a pretreatment with tumour necrosis alpha (TNFα) and interferon gamma (IFNγ) was administered. The integrity of the intercellular contacts was analyzed by immunofluorescence of junctional proteins. EDTA treatment alone caused breaks and mislocalization of intercellular contact proteins (Supplementary Fig. S4). The effect of Fe-EDTA and Ca-EDTA was comparable, whereas Na-EDTA detached the whole cell monolayer at the same concentrations. TNFα + IFNγ alone had a similar effect compared to EDTA, and enhanced the disruption of the epithelial barrier components by EDTA. We next assessed the integrity of the barrier by measuring paracellular FITC-dextran permeability in T84 cell monolayers. The results showed an additive effect of inflammation and EDTA on increased permeability (Fig. 3a). To further confirm the direct disruption of epithelial barrier by EDTA, we studied the effect of topical EDTA application in vivo using endomicroscopy. Healthy mice received two applications of Na-EDTA rectally for 10 min. Upon fluorescein injection, endomicroscopy showed increased permeability of the intestine for fluorescein. (Fig. 3b,c). Electron microscopy of samples obtained from these animals demonstrated the appearance of gaps on the lateral cell-to-cell contact surface that rarely reached the luminal or the basolateral surface, corresponding to disrupted AJs (Fig. 3d,e). There were few desmosomes present, which were intact. The adhesion to the basal membrane showed no abnormalities. Altogether, these in vitro and in vivo studies demonstrated a direct effect of EDTA on epithelial barrier disruption.

**Figure 4:**
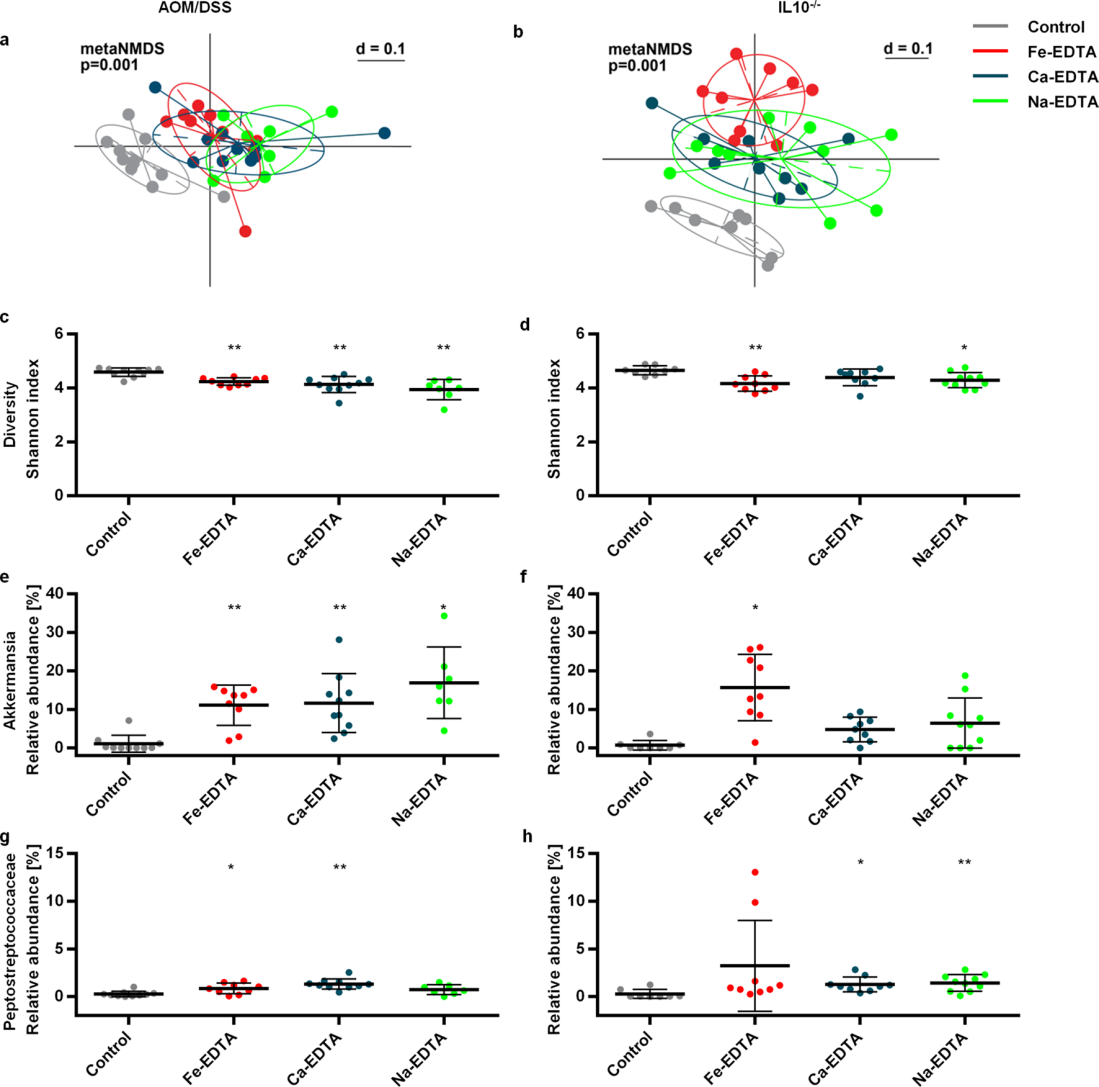
EDTA compounds induce dysbiosis in both the AOM/DSS and IL10^−/−^ models. **(a**,**b**) Meta-NDMS plots demonstrating significantly different microbial composition in control animals versus EDTA-treated animals in the AOM/DSS (**a**) and IL10^−/−^ model (**b**). (**c,d**) Shannon index as a measure of microbial diversity in the AOM/DSS (**c**) and IL10^−/−^ model (**d**). (**e–j**) Relative abundance of the most abundant and differentially represented microbial genera Akkermansia (**e,f**) and Peptostreptococcaceae (unknown genus) (**g,h**) in the AOM/DSS (**e,g**) and IL10^−/−^ model (**f,h**). Error bars represent standard deviations. Asterisks (*: p < 0.05; **: p < 0.01; ***: p < 0.001) denote statistically significant results compared to the control group.

Next we examined the composition of the bacterial flora in EDTA-treated mice by bacterial 16S-rRNA gene amplicon sequencing. The microbiome analysis revealed clear separation of EDTA groups in both AOM/DSS and IL10^−/−^ models, combined with a decrease in diversity (Fig. 4). The most notable change was a > 10-fold increase in the abundance of *Akkermansia muciniphilia*, a mucin-degrading bacterium of the phylum Verrucomicrobia. *A. muciniphilia* is typically reduced in patients with active IBD^25^, although some studies associate it with proinflammatory^26^ and procarcinogenic properties^27^. This is one of the first species able to colonize the intestine after fecal microbiota transplantation^28,29^, therefore, its increase might simply reflect a regeneratory state. A slight increase in *Peptostreptococcae* was also present. *Peptostreptococcus anaerobius* has previously been attributed procarcinogenic properties^30^, the exact functional consequence of our finding remains unknown.

## Discussion

Our results demonstrate that EDTA is toxic to the intestine when inflammation is present in doses that were not expected to cause any adverse effects. The addition of EDTA compounds to the food strongly enhances intestinal inflammation and colorectal carcinogenesis in two biologically different models of IBD. We show that EDTA disrupts various components of the intestinal barrier and increases intestinal permeability. This effect is also present in healthy animals and is likely massively aggravated in the presence of inflammation, leading to impaired wound healing and perpetuation of the inflammatory cascade. Dysbiosis is also induced by EDTA and it may contribute to the toxic effects.

No noticeable toxicity of EDTA in very high doses has been shown in multiple safety testings in healthy animals, leading to the recommended safety doses for human use. The ADI of 1.9 mg EDTA/kg bw recommended by the Joint FAO/WHO Expert Committee on Food Additives (JECFA) in 1973 for humans^31^ is derived from the NOAEL for rats of 250 mg EDTA/kg bw from the study by Oser et al.^32^, where 250 mg EDTA/kg bw was the highest concentration used; in other studies doses up to 2500 mg/kg bw have shown no toxicity^5,33^. Diarrhea as an adverse event has not been described in doses below 250–1000 mg/kg bw^6,34,35^, which is about three-fold higher than the highest dose in our study. Although EDTA is capable of disrupting the intestinal barrier in healthy animals as demonstrated by our experiments (Fig. 3b,c), the magnitude of the effect is obviously insufficient to cause a detectible clinical phenotype. Only for Fe-EDTA, several studies in colitis models have demonstrated an increase in intestinal inflammation and colitis-associated carcinogenesis; but the effect was attributed to iron and not EDTA^8,36^. In line of our findings, Constante et al. noted increased intestinal inflammation in DSS-treated mice with Fe-EDTA but not other iron compounds, concluding that intestinal toxicity might be specific to Fe-EDTA^37^. None of these studies have pointed to EDTA as the specific toxic moiety. However, while the compound seems safe in a healthy intestine, our results show that this is clearly not the case in the presence of gut inflammation. Unfortunately, there is no human data in the setting of IBD, infectious diarrhea or colorectal cancer, which, in the light of our study are specific risk populations.

Our study has several limitations. It utilizes only animal and cell culture models, and no data are available on human exposure to EDTA. The disruption of epithelial barrier components in both colitis models is not as apparent as in the healthy mice or in cultured epithelial cell monolayers. This is most likely due to the time point of sacrifice that was rather late after the initial inflammatory stimulus (in order to better observe tumour development). It remains not entirely clear how intestinal inflammation enhances EDTA toxicity, as similar changes are observed in healthy mice after a single short-term EDTA exposure. We hypothesize that a healthy mucosal barrier is more resistant to EDTA, and that a disruption of the barrier components by inflammation exposes deeper and otherwise protected mucosal structures to EDTA and facilitates translocation of commensal intestinal bacteria. Disruption of the barrier components (mucin^38,39^, tight junctions^40^, desmosomes^41,42^, hemidesmosomes^43^) by genetic defects or by immunological or chemical methods causes intestinal inflammation by itself, which makes it impossible to study these two factors independently in vivo. A direct effect of EDTA in promoting dysbiosis and therefore inflammation is also possible, as EDTA alters the stability of bacterial cell walls, delays microorganism growth and prevents adhesion by its sequestering action on divalent cations^44^. It remains unclear whether the increased colitis-associated carcinogenesis is solely secondary to inflammation or whether EDTA also has direct procarcinogenic properties. Scheers et al. demonstrated that Fe-EDTA may promote the proliferation of CaCo-2 and Hutu-80 cancer cells by activating Erk via increased levels of amphiregulin and EGFR but not via the Wnt pathway^45^. Studies have also proposed that EDTA may disrupt DNA stability by interfering with DNA-bound proteins by chelating Zn^2+^, possibly also Ca^2+^ and Mg^2+^^6^; the functional relevance of such chelation remains unknown. It remains unexplored whether the chelating action of EDTA can influence the intracellular redox balance, thus promoting direct DNA damage.

Nevertheless, the relevance of our findings remains high. EDTA is widely used and very stable. It is detected in most large rivers and even found in the drinking water in concentrations up to 30 µg/l^4^. The gastrointestinal (GI) tract would be inevitably exposed to EDTA especially by water together with its use in foods, pharmaceutics, cosmetics and household chemicals. Because of differences in local regulations and practices, the extent of exposure to EDTA is likely to vary from country to country. According to our results, due to the presumed EDTA’s toxicity in a specific population (IBD), a lowering and re-determining of the ADI is warranted. Also, specific recommendations in individuals with GI diseases such as IBD, irritable bowel syndrome, GI cancer or infectious diarrhea should be issued. It would be important to address the exposure to EDTA in healthy individuals and in patients with the above mentioned conditions in further studies, although studying dietary factors and linking them to a disease phenotype is notoriously problematic due to inconsistent exposure over time, ethical issues preventing a randomized design, difficult data collection and large number of subjects required for a cohort study. This study also highlights the shortcoming of the way food additive testing is done only in healthy animals. Other food additives and dietary agents have shown relevant intestinal toxicity in the presence of intestinal inflammation that was not apparent in healthy animals, such as emulsifiers^38^, TiO_2_^46^, or most recently polyunsaturated fatty acids^47^. Within the healthy human population itself, disruption of GI barrier function is common and linked to numerous GI conditions as mentioned above. We propose to remove EDTA from ingested substances and to include intestinal inflammatory models in future safety testing.

## Conclusion

We demonstrate a previously unrecognized intestinal toxicity of EDTA, a chelator used as a food additive and in pharmaceuticals among numerous other applications. EDTA salts induce massive intestinal inflammation and increased colorectal carcinogenesis in biologically different animal and cell culture models of inflammatory bowel disease at doses that are comparable to human use. We propose that the disruption of the epithelial barrier function may be the mechanism of the observed effect. Interestingly, this toxicity is not evident in healthy animals and therefore has been missed by regulatory safety testing. We therefore suggest the inclusion of intestinal inflammatory models in safety testing procedures for food additives as a strategy to detect otherwise unrecognizable toxicity in the intestine as a primary organ of exposure.

## Methods

### Animals

All experiments were performed in accordance with the Austrian and European law, defined by the Good Scientific Practice guidelines of the Medical University Vienna (animal ethics approval number: BMWFW-66.009/0072-WF/V/3b/2016 and BMWF-66.009/0062-/3b/2013). For all experiments, six week old male and female mice were used. C57BL/6 mice were purchased from Janvier, C57BL6 IL10^−/−^ mice were originally a kind gift from Dr. Terrence Barrett (University of Kentucky, Lexington, KY, USA) and bred under specific pathogen-free conditions at the Division of Laboratory Animal Science and Genetics, Department of Biomedical Research at the Medical University of Vienna (Himberg, Austria) and used at an age of 6 weeks. All experiments were performed under conventional husbandry at the facilities of the Department of Biomedical Research at the Medical University of Vienna (Vienna, Austria). At arrival, the animals were distributed to cages with maximum of four animals per cage; the stratification was according to sex and weight without a dedicated randomization procedure. Prior to the start of the experiments, the animals had a two weeks adaption phase, where no experimental diets were administered. Animals received food and tap water (unless otherwise mentioned) ad libitum. The experimental diets were commenced as specified for every experiment. The composition of the diets is summarized in Supplementary Tables S1 and S4. The researchers involved were not blinded to the group allocation. The 173 mg EDTA/kg bw dose was chosen as the NOAEL dose for Fe-EDTA according to EFSA^5^. The 21 mg EDTA/kg bw dose was chosen to represent the ADI in humans and is derived according to the formula ADI = NOAEL × 0.01 × 12.3, where 0.01 is the overall default uncertainty factor to account for inter- and intraspecies variability as recommended by EFSA^48^, and 12.3 is the body surface area-based metabolic weight conversion factor between mice and humans^49^. The conversion between mg/kg bw and mg/kg chow was performed using the quotient 0.149, also recommended by EFSA^48^. The ADI for humans as set by JEFCA is 1.9 mg EDTA/kg bw (or 2.5 mg Ca-EDTA/kg bw), corresponding to 23.37 mg EDTA/kg bw for mice and is slightly higher than the one we used (21 mg/kg bw)^31^.

For the AOM/DSS model^15,16^, azoxymethane (Sigma Aldrich, St. Louis, MO, USA) was administered at a concentration of 7.5 mg/kg bw intraperitoneally at a time point specified for every experiment. Dextran sodium sulfate (MP Biomedicals, Eschwege, Germany) was given with the drinking water at a concentration of 1.5% weight/volume for five days. The timepoint of each DSS cycle is denoted at the description of the corresponding experiments. During a subsequent recovery phase, the animals received tap water.

For the IL10^−/−^ model^17^, colitis induction and synchronization was done using piroxicam (Sigma Aldrich) at 200 ppm with the chow^50^ for one cycle of 8 days (Fig. 1) or two cycles of five days with a recovery phase of 4 days between cycles (Fig. 2).

Throughout the animal experiments, the mice were weighed at least once per week, and their stool was examined for consistency and the presence of overt or occult blood (using a guaiac test, Haemoccult, Beckman Coulter). These variables were used to obtain a clinical disease activity score (DAI)^51^ (Supplementary Table S2).

At the end of the experiment, the mice were sacrificed by cervical dislocation after anesthesia with xylazine and ketamine.

### Histology and immunohistochemistry

The intestines were collected, flushed and prepared using the Swiss roll technique^52^ and subsequently fixed in phosphate buffered formalin 10%. A subset of the intestines was not flushed but fixed including the content using the mucus preserving methacarn solution (methanol 60%, chloroform 30%, glacial acetic acid 10%). Five µm cuts were prepared after standard dehydration and paraffin embedding procedures. For the examination of colitis activity and presence of tumours, hematoxylin and eosin stains were performed using a standard method. The intestines were then examined under a light microscope (Olympus BX51 microscope with an Olympus DP73 microscope camera, Tokyo, Japan). A histological colitis activity index (HAI)^53,54^ (Supplementary Table S3) was obtained. The intestinal tissue was also examined for the presence of tumours, and the area of each tumour was measured using the CellSens Dimension Version 1.17 software (Olympus, https://www.olympus-lifescience.com/de/software/cellsens/). The presence of invasiveness (i.e. breakthrough through the mucularis mucosae) was noted; a dysplasia grading was not performed. The examiners (TA and AC) were not blinded for the group allocation. The following variables were calculated: total tumour burden (i.e. sum of all tumour areas per mouse), mean tumour size, tumour multiplicity (number of tumours per mouse) and invasive tumour multiplicity.

The intactness of the mucus layer was evaluated using methacarn-fixed intestines. Periodic acid Schiff’s base (PAS) mucus stain was performed using a standard method, and the intactness of the layer was examined and graded 0%-100% under a light microscope.

The intactness of intercellular contacts was examined by immunohistochemistry, which was performed using a standard method. The antibodies used are listed in Supplementary Table S5. The intensity of the stain was graded as 0 (no expression), 0.5 (weak expression) and 1 (strong expression), multiplied with the estimated relative area with the corresponding staining intensity, thus resulting in an immunoreactivity score ranging from 0 to 100%. The examiner (GD) was blinded for the group allocation by opacifying the slide labeling.

### Measurement of fecal EDTA content

Stool samples from a subset of animals were collected immediately post mortem, snap frozen in liquid nitrogen and stored at − 80 °C. The stool samples were dried at 105 °C and homogenized mechanically, by sonification and shaking for 1 h in a Fe-complexing solution (20 mg Fe(III) sulfate diluted to 100 ml with a 1.5 mM sulfuric acid solution; all reagents from Sigma Aldrich). The centrifuged supernatant was subjected to high performance liquid chromatography using a HPLC-System 1260 with diode array detector (Agilent Technologies, Santa Clara, CA, USA) and a Hamilton PRP-X100 anion exchange column (10 µm particle size, Sigma Aldrich). The main peak of EDTA was measured at 300 nm using diode array detection and G2170AA Rev. B.04.03 software (Agilent, https://www.agilent.com). The analyses were performed at the Chemcon laboratory (Vienna, Austria).

### Paracellular permeability assay

T84 cells (source: ATCC, Manassas, VA, USA) were grown in Dulbecco’s modified Eagle’s medium-F12 (Gibco, Thermo Fisher Scientific, Waltham, MA, USA) supplemented with 10% fetal bovine serum (Biochrom, Berin, Germany). 1 × 10^5^ cells were plated on 24-well polystyrene transwells (0.4-μm pore size; Costar, Corning, NY, USA) for 14 days to form intact monolayers as described previously^23^. To mimic inflammation, the monolayers were pretreated with tumour necrosis factor alpha (TNFα, 50 ng/ml, Miltenyi Biotec, Bergisch Gladbach, Germany) and interferon gamma (IFNγ, 50 ng/ml, eBioscience, Thermo Fischer Scientific) for 1 h and then with EDTA compounds (Fe-EDTA at 4 mM, Ca-EDTA at 4 mM or Na-EDTA at 0.625 mM, corresponding to 1168 mg EDTA/l or 182.5 mg EDTA/l) for 3 h. A lower dose of Na-EDTA compared to the other compounds had to be used, as higher concentrations led to an immediate detachment of the cells. 10-kDa fluorescein isothiocyanate-labeled dextran (FITC-dextran, 1 mg/ml, Sigma Aldrich) was then applied on the luminal monolayer surface, and the supernatant from the basolateral surface was obtained after 60 min. The concentration of the paracellularly leaked FITC-dextran was measured by fluorescence emission on a Chameleon Counter (HVD Life Sciences, Vienna, Austria) at 485 nm/535 nm. Experiments were performed in biological triplicates. Subsequently, immunofluorescence for intercellular contact proteins was performed using a standard protocol and antibodies as noted in Supplementary Table S5 at a dilution of 1:200.

### Confocal laser endomicroscopy

Healthy 6 weeks old C57BL/6 male mice (n = 3 per group) were used. One day prior to the experiment, a bowel preparation solution (2.6 g NaCl, 13.5 g glucose, 1.5 g KCl, 2.9 g trisodium citrate, 34.5 g polyethylenglycol 35000 (all from Sigma Aldrich), distilled water to a total volume of 1 l) was given instead of drinking water. The animals were anesthetized using ketamin 100 mg/kg and xylazine 12 mg/kg intraperitoneally. After removing any stool rests in the sigmoid colon by flushing under visualization using a miniature sigmoidoscope (Karl Storz, Tuttlingen, Germany; airpump from Eheim, Deizisau, Germany), EDTA or saline were applied via the working channel of the endoscope. After administration of fluorescein (0.05 mg/g bw intraperitoneally, Fluorescite, Alcon Ophthalmika GmbH, Vienna, Austria), two rectal applications of 2 ml Na-EDTA (1 mM, corresponding to 292 mg EDTA/l) or saline for 10 min were given. A Cellvizio Confocal Miniprobe (Mauna Kea Technologies, Paris, France) was then introduced per anum. Confocal laser endomicroscopy of the left colon was then performed.

### Electron microscopy

After fixation in Karnovsky solution (paraformaldehyde 4%, glutaraldehyde 5%), tissues were postfixed in 1% osmium ferrohexacyanoferrate II, dehydrated in ethanol and propylene oxide and embedded in Epon resin. Ultrathin sections of 70 nm were made on a Leica-Ultracut- EM-UC7. For contrast enhancement, the ultrathin sections were stained in 2% uranyl acetate and 1% lead citrate. Transmission electron microscopy was then performed on a TEM Jeol 1400 Plus (Jeol, Tokyo, Japan) at 60 kV, pictures were taken with Quemesa Camera in RADIUS v. 2.0 software (Emsis Ltd., Muenster, Germany, https://www.emsis.eu).

### Microbiome analysis

Stool samples were obtained immediately post mortem and snap frozen in liquid nitrogen. DNA was extracted using the standard QIAamp DNA stool mini kit protocol (Qiagen, Venlo, Netherlands) modified by an initial bead-beating-step (Lysing Matrix E, MP Biomedicals). Amplicon sequencing of the V3V4 16S-region was performed using standard Illumina MiSeq protocols^55^. Reads were processed using DADA2^56^ and SINA^57^. For the analysis of sample similarity modified Rhea scripts were used^58^.

### Statistical analysis

All statistical analyses were performed using SPSS Version 23 (IBM, Armonk, NY, USA), GraphPad Prism Version 6.01 (GraphPad Software Inc, San Diego, CA, USA), or R Version 3.6.2 (R Core Team, R Foundation for Statistical Computing, Vienna, Austria, 2019, https://www.R-project.org). Sample size calculations for the animal experiment described in Fig. 1 (a pilot experiment) were based on the assumption of doubling the tumour multiplicity with the iron compounds compared to control, using a power of 0.8 and significance of 0.05. For the experiment depicted in Fig. 2, sample size calculations were based on the estimates for total tumour burden from the experiment from Fig. 1 using a power of 0.9 and a significance of 0.05. Bonferroni-corrected t-tests were utilized for the calculation of the sample size. The other presented parameters were considered as secondary endpoints. Results from animals that died prematurely have not been used in the final analysis. The sample size for the further experiments was empirically set at 3, as they were considered pilot experiments. For all final analyses, non-parametric Kruskal–Wallis ANOVA with Dunn’s post-hoc tests were used for comparisons of more than two groups, and Mann–Whitney U-test for comparisons between two groups. For the microbiome analysis, generalized UniFrac distances were visualized using non-metric multi-dimensional scaling (NMDS). Cluster significance was assessed using permutational multivariate analysis of variance. Testing for significant differences in diversity and bacterial abundances was performed using Kruskal–Wallis Rank Sum Test with Benjamin-Hochberg method for correction for multiple comparisons. All tests were performed as two-tailed tests. A p-value below 0.05 was considered statistically significant for all tests.

The datasets generated and analyzed during the current study are available from the corresponding author on reasonable request.

The study was carried out in compliance with the ARRIVE guidelines.

## Supplementary Information

Supplementary Figure S1.

Supplementary Figure S2.

Supplementary Figure S3.

Supplementary Figure S3.

Supplementary Figure S4.

Supplementary Table S1.

Supplementary Table S2.

Supplementary Table S3.

Supplementary Table S4.

Supplementary Table S5.

Supplementary Legends.

## Abbreviations

ADI: Acceptable daily intake
AJ: Adherens junction
AOM: Azoxymethane
bw: Body weight
DAI: Disease activity index
DSS: Dextran sodium sulfate
EDTA: Ethylenediaminetetraacetate
EFSA: European Food Safety Authority
FAO: Food and Agriculture Organization
GI: Gastrointestinal
HAI: Histological activity index
IL10: Interleukin 10
IBD: Inflammatory bowel disease
IFNγ: Interferon gamma
JEFCA: Joint FAO/WHO Expert Committee on Food Additives
NOAEL: No observed adverse effects level
TJ: Tight junction
TNFα: Tumour necrosis factor alpha
WHO: World Health Organization
