# Supplementary figures and images for: The food additive EDTA aggravates colitis and colon carcinogenesis in mouse models
Source: Sci Rep. 2021 Mar 4;11:5188. doi: 10.1038/s41598-021-84571-5 (PMC7933154; doi:10.1038/s41598-021-84571-5)

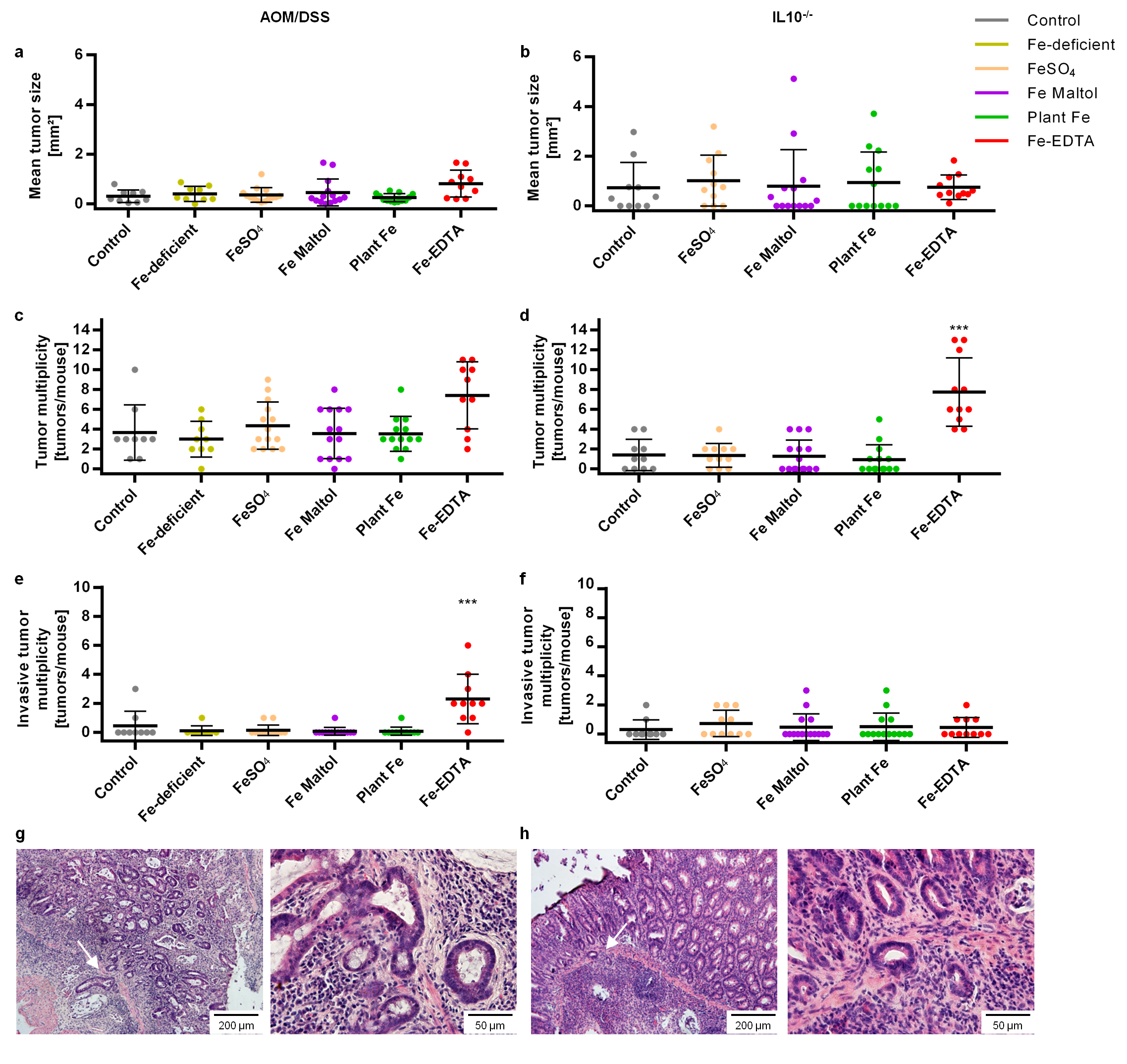

Supplement: Supplementary file 1 — Supplementary Figure S1. [file 41598_2021_84571_MOESM1_ESM.tif]

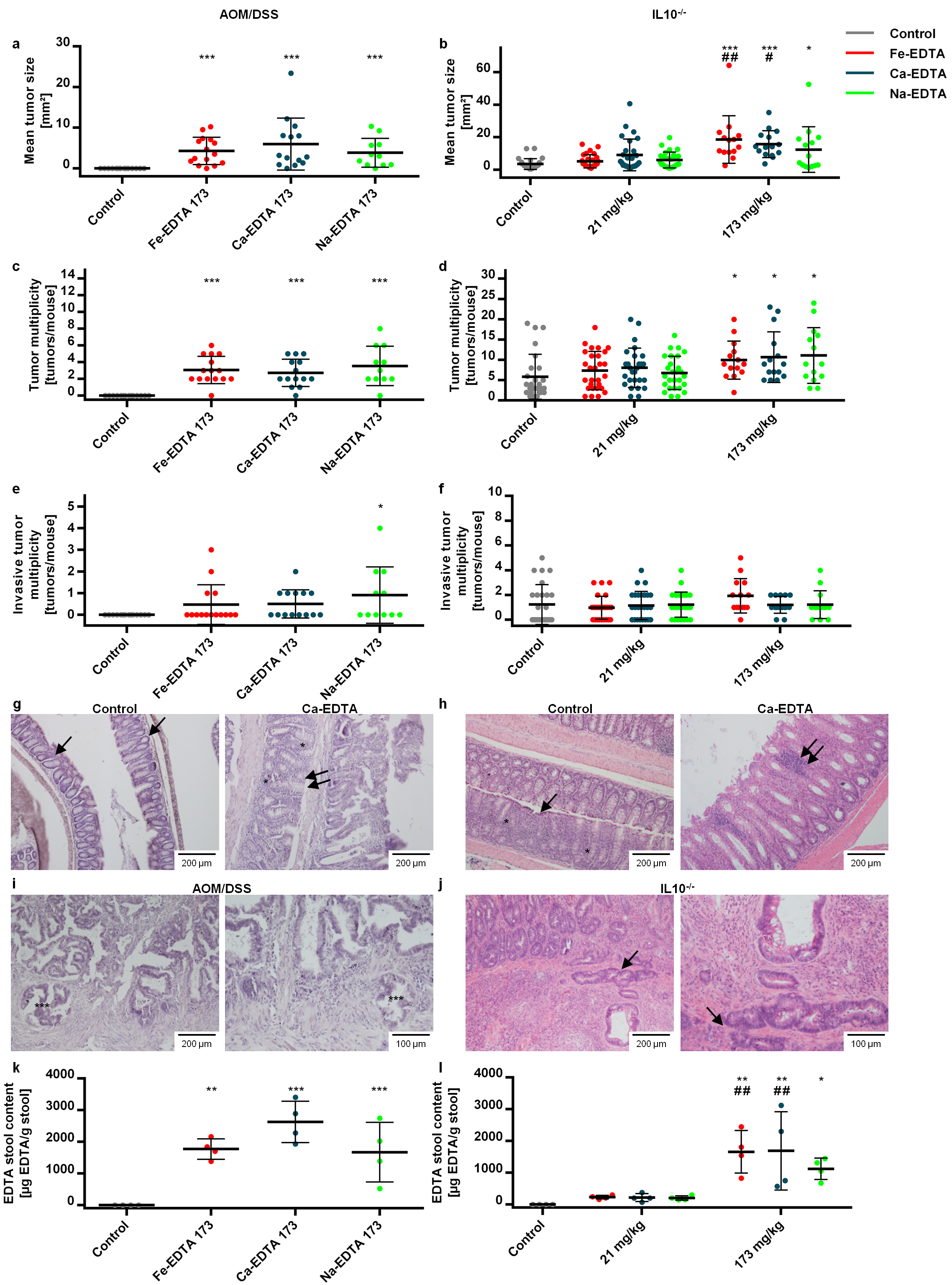

Supplement: Supplementary file 2 — Supplementary Figure S2. [file 41598_2021_84571_MOESM2_ESM.tif]

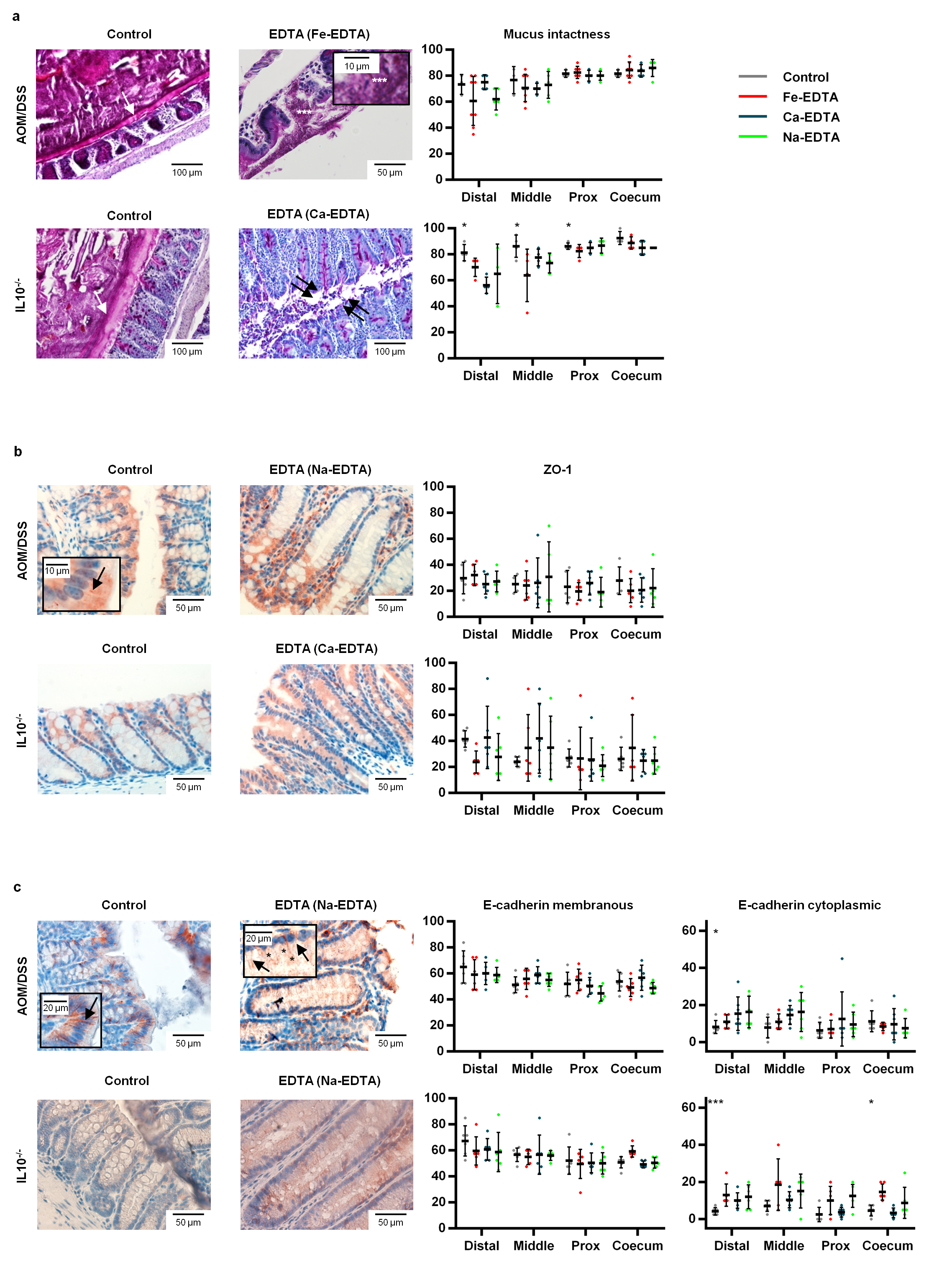

Supplement: Supplementary file 3 — Supplementary Figure S3. [file 41598_2021_84571_MOESM3_ESM.tif]

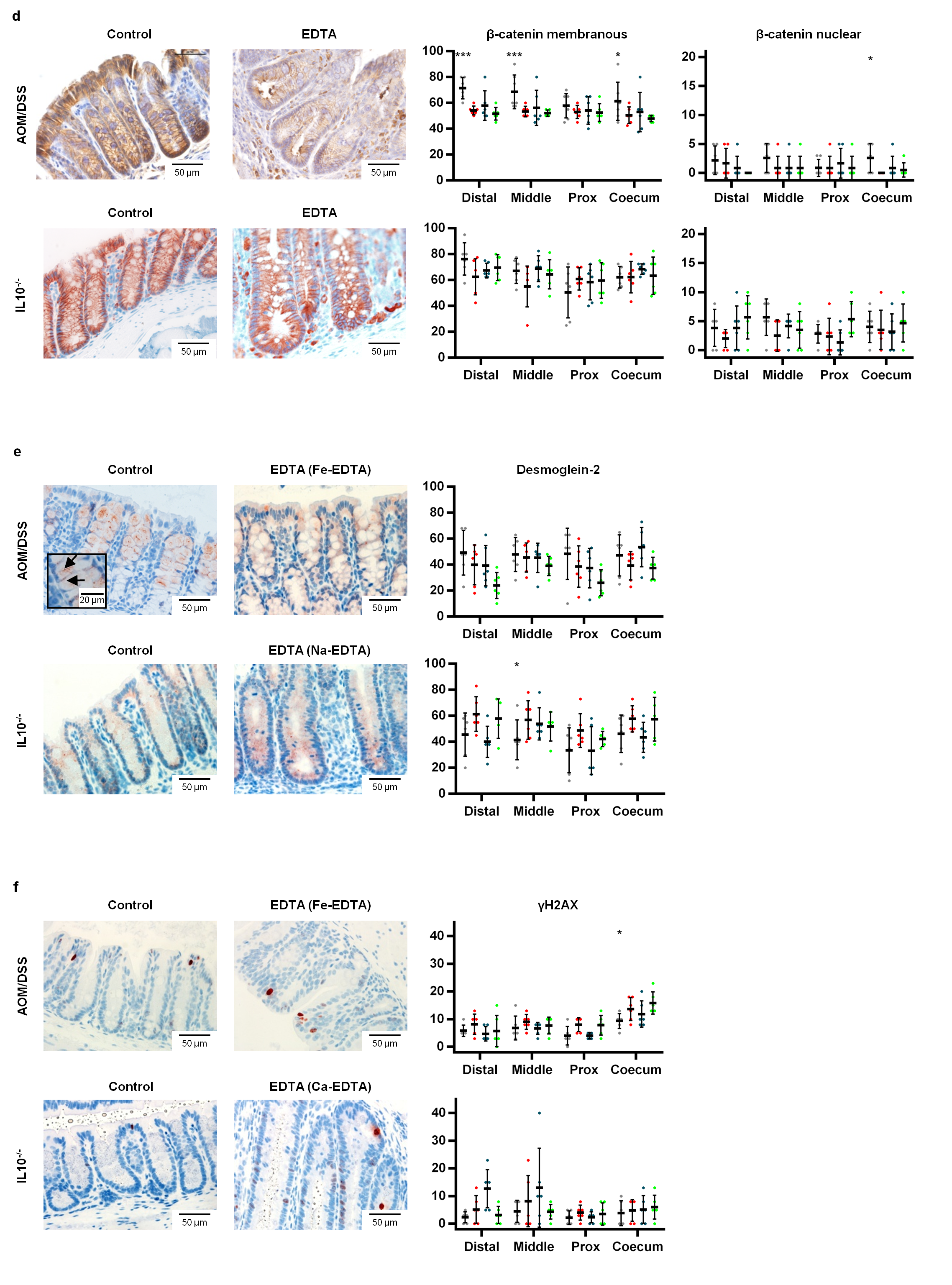

Supplement: Supplementary file 4 — Supplementary Figure S3. [file 41598_2021_84571_MOESM4_ESM.tif]

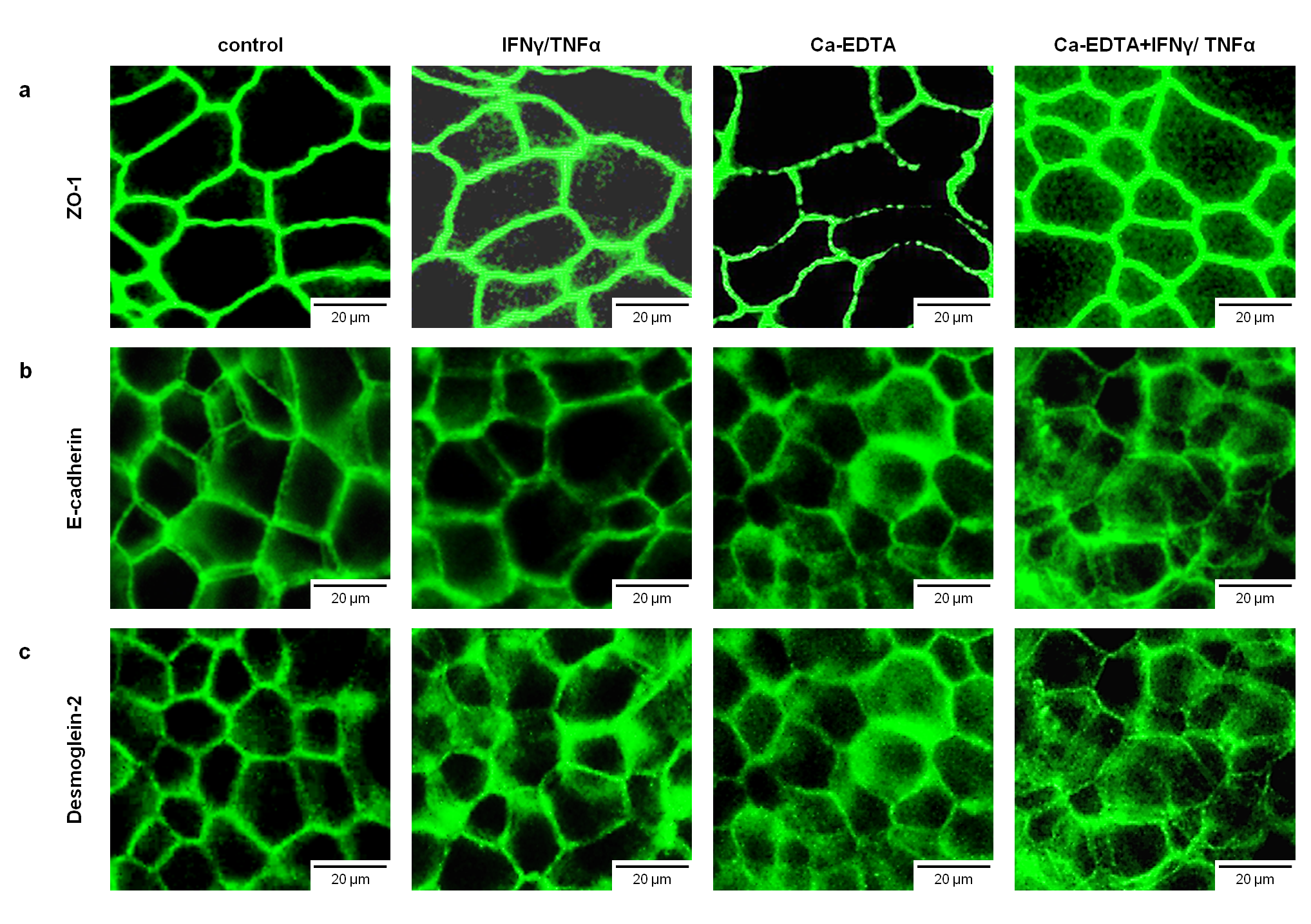

Supplement: Supplementary file 5 — Supplementary Figure S4. [file 41598_2021_84571_MOESM5_ESM.tif]
