# Supplementary material for: The food additive EDTA aggravates colitis and colon carcinogenesis in mouse models
Source: Sci Rep. 2021 Mar 4;11:5188. doi: 10.1038/s41598-021-84571-5 (PMC7933154; doi:10.1038/s41598-021-84571-5)
Supplement: Supplementary file 6 — Supplementary Table S1. [file 41598_2021_84571_MOESM6_ESM.docx]

**Extended Data Table 1. Experimental diet composition for the experiment displayed in Figure 1**

| Group | Compound ^†^ | Source | Elemental Fe [mg]/  kg chow | Compound [mg]/  kg chow |
| --- | --- | --- | --- | --- |
| Control | Fe(II) sulfate | Sigma Aldrich  (St. Louis, MO, USA) | 45 | 223 |
| Fe deficient | none | none | < 10 | none |
| FeSO_4_ | Fe(II) sulfate | Sigma Aldrich  (St. Louis, MO, USA) | 450 | 2230 |
| Fe maltol | Fe (III) maltol | AoP Orphan  (Vienna, Austria) | 450 | 3475 |
| Plant Fe | Curry leaf extract | Biogena Naturprodukte Ltd (Salzburg, Austria) | 450 | 11539 |
| Fe-EDTA | EDTA-Fe(III)Na.3H_2_O | Sigma Aldrich  (St. Louis, MO, USA) | 450 | 2958 (i.e. 351 mg EDTA/kg bw) |

^†^ Base chow: C1038 (< 10 mg elemental Fe/kg chow) (Altromin, Lage, Germany)
