# Supplementary material for: The food additive EDTA aggravates colitis and colon carcinogenesis in mouse models
Source: Sci Rep. 2021 Mar 4;11:5188. doi: 10.1038/s41598-021-84571-5 (PMC7933154; doi:10.1038/s41598-021-84571-5)
Supplement: Supplementary file 7 — Supplementary Table S2. [file 41598_2021_84571_MOESM7_ESM.docx]

**Extended Data Table 2. Disease activity index (DAI)**

| Score | Weight loss (%) | Stool consistency | Blood in stools |
| --- | --- | --- | --- |
| 0 | < 1% | normal | none |
| 1 | 1-5% |  |  |
| 2 | 5-10% | loose stools | guaiac positive |
| 3 | 10-15% |  |  |
| 4 | >15% | diarrhea | visible blood |

DAI = (weight loss score + stool consistency score + blood in stools score)/3 (Hamamoto et al, Clin Exp Immunol 1993)
