# Supplementary material for: The food additive EDTA aggravates colitis and colon carcinogenesis in mouse models
Source: Sci Rep. 2021 Mar 4;11:5188. doi: 10.1038/s41598-021-84571-5 (PMC7933154; doi:10.1038/s41598-021-84571-5)
Supplement: Supplementary file 8 — Supplementary Table S3. [file 41598_2021_84571_MOESM8_ESM.docx]

**Extended Data Table 3. Histological activity index (HAI)**

| Score | Inflammatory infiltrate | Epithelium loss | Hyperplasia |
| --- | --- | --- | --- |
| 0 | none | none | none |
| 1 | mild, limited to mucosa | loss of up to 1/3 of crypts | minimal |
| 2 | moderate, in mucosa and submucosa | loss of up to 2/3 of crypts | mild |
| 3 | severe, with obliteration of mucosal architecture, erosions and/or crypt abscesses | lamina propria covered with a single cell layer of epithelium | moderate |
| 4 | level 3 changes plus ulceration | complete loss of epithelium | marked |

HAI = (inflammatory infiltrate score + epithelium loss score + hyperplasia score) /3 (modified from from Cooper et al, Lab Invest 1993 and Erben et al, Int J Exp Pathol 2014)
