# Supplementary material for: The food additive EDTA aggravates colitis and colon carcinogenesis in mouse models
Source: Sci Rep. 2021 Mar 4;11:5188. doi: 10.1038/s41598-021-84571-5 (PMC7933154; doi:10.1038/s41598-021-84571-5)
Supplement: Supplementary file 9 — Supplementary Table S4. [file 41598_2021_84571_MOESM9_ESM.docx]

**Extended Data Table 4. Experimental diet composition for the experiment displayed in Figure 2**

| Group | Compound ^†^ | Trade name ^‡^ | mg EDTA/  kg bw | mg compound/  kg bw | mg EDTA/  kg chow | mg compound/  kg chow |
| --- | --- | --- | --- | --- | --- | --- |
| control | none | none | 0 | 0 | 0 | 0 |
| Fe-EDTA 173 | EDTA-FeNa.3H_2_O | Ferrazone XF ® | 173 | 250 | 1161 | 1678 |
| Ca-EDTA 173 | EDTA-CaNa_2_.2H_2_O | Solvitar ® | 173 | 243 | 1161 | 1630 |
| Na-EDTA 173 | EDTA-Na_2_H_2_.2H_2_O | Dissolvine Na2-P ® | 173 | 220 | 1161 | 1479 |
| Fe-EDTA 21 | EDTA-FeNa.3H_2_O | Ferrazone XF ® | 21 | 31 | 143 | 206 |
| Ca-EDTA 21 | EDTA-CaNa_2_.2H_2_O | Solvitar ® | 21 | 30 | 143 | 200 |
| Na-EDTA 21 | EDTA-Na_2_H_2_.2H_2_O | Dissolvine Na2-P ® | 21 | 27 | 143 | 182 |

^†^ Base chow: R/M-H diet (ssniff Spezialdiäten Ltd, Soest, Germany)

^‡^ All compounds were purchased from AkzoNobel, Amsterdam, Netherlands
