# Supplementary material for: The food additive EDTA aggravates colitis and colon carcinogenesis in mouse models
Source: Sci Rep. 2021 Mar 4;11:5188. doi: 10.1038/s41598-021-84571-5 (PMC7933154; doi:10.1038/s41598-021-84571-5)
Supplement: Supplementary file 10 — Supplementary Table S5. [file 41598_2021_84571_MOESM10_ESM.docx]

**Extended Data Table 5. Antibodies used for immunohistochemistry and immunofluorescence analyses**

| Target | Antibody | Dilution for immunohistochemistry ^†^ |
| --- | --- | --- |
| ZO-1 | #617300, Thermo Fisher Scientific, Waltham, MA, USA | 1:250 |
| E-cadherin | #610182, BD Biosciences, Franklin Lakes, NJ, USA | 1:1000 |
| β-catenin | #610153, BD Biosciences, Franklin Lakes, NJ, USA | 1:1000 |
| Desmoglein-2 | #ab150372, Abcam, Cambridge, UK | 1:1500 |
| γH2AX | #9718, Cell Signalling Technology, Danvers, MA, USA | 1:500 |

^†^ for immunofluorescence, all antibodies were diluted 1:200
