# Supplementary material for: The food additive EDTA aggravates colitis and colon carcinogenesis in mouse models
Source: Sci Rep. 2021 Mar 4;11:5188. doi: 10.1038/s41598-021-84571-5 (PMC7933154; doi:10.1038/s41598-021-84571-5)
Supplement: Supplementary file 11 — Supplementary Legends. [file 41598_2021_84571_MOESM11_ESM.docx]

**The food additive EDTA aggravates colitis and colon carcinogenesis in mouse models Authors:** ^1^Rayko Evstatiev, ^1^Adam Cervenka, ^1^Tina Austerlitz, ^1^Gunther Deim, ^1^Maximilian Baumgartner, ^2^Andrea Beer, ^1^Anita Krnjic, ^1^Christina Gmainer, ^1^Michaela Lang, ^1^Adrian Frick, ^2^Helga Schachner, ^1^Vineeta Khare and ^1^Christoph Gasche^*^

^1^Department of Internal Medicine III, Division of Gastroenterology and Hepatology, Medical University of Vienna, Waehringer Guertel 18-20, A-1090 Vienna, Austria

^2^ Department of Pathology, Medical University of Vienna, Waehringer Guertel 18-20, A-1090 Vienna, Austria

***Correspondence**: Christoph Gasche, M.D. Division of Gastroenterology and Hepatology, Internal Medicine 3, Medical University of Vienna, Waehringer Guertel 18-20A-1090 Vienna, Austria. Phone +4314040047640; +431 4040047350 (fax), email: christoph.gasche@meduniwien.ac.at

**Extended data figure legends**

**Extended Data Figure 1. Tumour characteristics in Fe-EDTA treated mice compared to other iron compounds.** **a** and **b**: Mean tumour size for the AOM/DSS (**a**) or IL10^-/-^ (**b**) models. **c** and **d**: Tumour multiplicity (i.e., number of tumours per mouse) for the AOM/DSS (**c**) or IL10^-/-^ (**d**) models. **e** and **f**: Invasive tumour multiplicity (i.e., number of invasive tumours per mouse) for the AOM/DSS (**e**) or IL10^-/-^ (**f**) models. **g** and **h**: Representative images of invasive tumours in Fe-EDTA-treated animals for the AOM/DSS (**g**) or IL10^-/-^ (**h**) models. Arrows show the point of invasion through the lamina muscularis mucosae. Error bars represent standard deviations. Asterisks (*: p < 0.05; **: p < 0.01; ***: p < 0.001) denote statistically significant results compared to the Fe-EDTA group.

**Extended Data Figure 2. Additional characteristics of inflammation, tumours and fecal EDTA content in mice treated with different EDTA compounds.** **a** and **b**: Mean tumour size for the AOM/DSS (**a**) or IL10^-/-^ (**b**) models. **c** and **d**: Tumour multiplicity (i.e., number of tumours per mouse) for the AOM/DSS (**c**) or IL10^-/-^ (**d**) models. **e** and **f**: Invasive tumour multiplicity (i.e., number of invasive tumours per mouse) for the AOM/DSS (**e**) or IL10^-/-^ (**f**) models. **g**: Microscopy images of hematoxylin-eosin-stained intestines from the AOM/DSS model showing inflammation in control and EDTA-treated animals (Ca-EDTA-treated animal chosen for representation). In control animals, mild crypt distortion and hyperplasia (single arrow) with a minimal increase in lamina propria cellularity can be seen, whereas EDTA-treated animals display pronounced architectural changes and hyperplasia (double arrows) combined with increased inflammatory infiltrate (asterisks). **h**: Microscopy images of hematoxylin-eosin-stained intestines from the IL10^-/-^ model demonstrating inflammation in control vs. EDTA-treated animals (Ca-EDTA shown). Increased inflammatory infiltrate (asterisk) and architectural changes (single arrow) in a control animal are marked. With EDTA, massive hyperplasia and inflammatory cell collection (lymphocyte aggregates; double arrow) are seen compared to control. **i** and **j**: Representative images of invasive tumours in Fe-EDTA-treated animals for the AOM/DSS (**i**) or IL10^-/-^ (**j**) models. Invasiveness is recognized by portions of the tumour located entirely in the lamina muscularis propria (three asterisks) or by a clear point of invasion through the lamina muscularis mucosae (single arrow). **k** and **l**: fecal EDTA content in dried stool samples in the AOM/DSS (**k**) or IL10^-/-^ (**l**) models. All EDTA doses are expressed in mg EDTA/kg bw. Error bars represent standard deviations. Asterisks (*: p < 0.05; **: p < 0.01; ***: p < 0.001) denote statistically significant results compared to the control group. Hashtags (#: p < 0.05; ##: p < 0.01; ###: p < 0.001) mark significant differences between both EDTA compound doses.

**Extended Data Figure 3. EDTA weakens various components of the epithelial barrier in the AOM/DSS and IL10^-/-^ models.** **a**: PAS stain visualizing the mucus layer. Single arrows denote intact mucus layer. Double arrows point to interrupted and/or missing mucus layer. Asterisks demonstrate bacterial invasion of the mucus layer. **b**: ZO-1-immunostaining as a marker for tight junctions. Single arrow points to focal membranous ZO-1 expression in the tight junctions. **c**: E-cadherin immunostaining as a marker for AJs. Arrows demonstrate membranous E-cadherin expression in the AJs, asterisks cytoplasmic E-cadherin. **d**: β-catenin immunostaining **e**: desmoglein-2 immunostaining as a marker of desmosomes. Arrows demonstrate focal membranous desmoglein-2 expression in the desmosomes. **f**: γH2AX immunostaining as a marker of double strand DNA breaks. All dot blots represent an immunoreactivity score (**b-f**) or mucus intactness score (**a**) ranging from 0% (no stain) to 100% (maximum staining intensity; see Methods for details). Error bars represent standard deviations. Asterisks (*: p < 0.05; **: p < 0.01; ***: p < 0.001) denote statistically significant comparisons between the pooled EDTA groups and the control group.

**Extended Data Figure 4. EDTA treatment of T84 cell monolayers disrupts intercellular contacts synergistically to inflammatory stimulus.** Results from Ca-EDTA-treated monolayers are shown as representation, as all EDTA compounds induced similar changes. **a**: ZO-1 staining as a marker of tight junctions. **b**: E-cadherin staining as a marker of AJs. **c**: Desmoglein-2 staining as a marker of desmosomes.
